# *Disjunctitermes insularis*, a new soldierless termite genus and species (Isoptera, Termitidae, Apicotermitinae) from Guadeloupe and Peru

**DOI:** 10.3897/zookeys.665.11599

**Published:** 2017-04-03

**Authors:** Rudolf H. Scheffrahn, Tiago F. Carrijo, Anthony C. Postle, Francesco Tonini

**Affiliations:** 1 Fort Lauderdale Research and Education Center, Institute for Food and Agricultural Sciences, 3205 College Avenue, Davie, Florida 33314, USA; 2 Centro de Ciências Naturais e Humanas, Universidade Federal do ABC, Rua Arcturus 03, Jardim Antares, 09606-070, São Bernardo do Campo, SP, Brazil; 3 P.O. Box 5473 Cairns Queensland 4870, Australia; 4 Center for Systems Integration and Sustainability, Michigan State University, 115 Manly Miles Building, 1405 S. Harrison Rd., East Lansing, MI 48823, USA

**Keywords:** Soil-feeder, taxonomy, barcode sequence, stochastic spread, overwater dispersal

## Abstract

*Disjunctitermes insularis* **gen. n. & sp. n.** is described from workers collected on Guadeloupe and in Peru and is the first soldierless termite found on a deep-water island. As with many soldierless and soil-feeding termite species, the enteric valve morphology is an essential diagnostic character of *D. insularis*. The *D. insularis* sequence cluster, derived from a barcode analysis with twelve other described genera of New World Apicotermitinae, is well resolved. Results of a stochastic dynamic spread model suggest that the occurrence of *D. insularis* on Guadeloupe may be the result of a pre-Colombian overwater dispersal event from mainland South America.

## Introduction

All New World species of the soil-feeding termite subfamily Apicotermitinae lack soldiers. The absence of the soldier caste has historically hindered the classification of this diverse group until the gradual adoption of worker digestive tract characters, especially the enteric valve (EV) morphology allowing for genus and species level discrimination (Bourguignon et al. 2016a, b). Recently, seven new genera, numerous new species, reassignments, and synonyms of Neotropical apicotermitines have been reported (Bourguignon et al. 2010, 2016a, Carrijo et al. 2015, Scheffrahn 2013, Scheffrahn et al. 2006).

Darlington (1992) listed 12 termite species, all wood feeders, on the island of Guadeloupe. In 1999, as part of our ongoing diversity study of the West Indies (Puerto Rico and the U.S. Virgin Is.: Scheffrahn et al. 2003a, Trinidad: Scheffrahn et al. 2003b, and the Bahamas: Scheffrahn et al. 2006) we also surveyed Guadeloupe and were astonished to collect numerous samples of a small soldierless termite species. In 2014, we collected a single sample of this same species in the Peruvian Amazon. We herein describe a new genus, *Disjunctitermes*, a single new species, *D. insularis*, discuss its remarkable distribution, and estimate its dispersal rate on Guadeloupe.

## Materials and methods

Workers were collected and preserved in 85% ethanol. External and internal dissections were suspended in Purell® Instant Hand Sanitizer in a plastic Petri dish and photographed using a Leica M205C stereomicroscope controlled by Leica Application Suite version 3.0 montage software. The EV was prepared by removing the entire worker P2 section in ethanol. Food particles were expelled from the P2 tube by pressure manipulation. The tube was quickly submerged in a droplet of PVA medium (BioQuip Products Inc.) which, by further manipulation, eased muscle detachment. The remaining EV cuticle was left intact or longitudinally cut, splayed open, and mounted on a microscope slide using the PVA medium. The EV was photographed with a Leica CTR 5500 compound microscope with phase-contrast optics using the same montage software. Terminology of the worker gut follows that of Sands (1998) and Noirot (2001).

Sequences of three specimens of *D. insularis* and twelve other samples of Neotropical Apicotermitinae (eight species in six genera, Table 1) were obtained by DNA extraction and PCR performed by the Canadian Centre for DNA Barcoding following standard high-throughput protocols (deWaard et al. 2008). The PCR employed the primers LepF1 and LepR1 (Hebert et al. 2003) which generated 622 to 652bp of the barcode region of the mitochondrial gene cytochrome c oxidase subunit 1 (COI). In addition, GenBank sequences from 20 neotropical Apicotermitinae (13 species in 10 genera), five non-apicotermitine Termitidae, and one Rhinotermitidae to root the tree (Table 1) were included in our analysis.

**Table 1.** Species used in the phylogeny, GenBank accession number, and UF collection code for those used in this study.

Species	GenBank	UF Code
*Amplucrutermes inflatus*	KT215783	
*Anoplotermes parvus*	HQ398187	
*Anoplotermes parvus*	HQ398189	
*Anoplotermes janus*	HQ398188	
*Anoplotermes janus*	KY683193	UF.FG208
*Anoplotermes janus*	KY683187	UF.PU827
*Anoplotermes banksi*	HQ398185	
*Anoplotermes banksi*	KT215785	
*Aparatermes spA*	KT215784	
*Aparatermes sivestrii*	KY683197	UF.TT2018
*Aparatermes silvestrii*	KY683190	UF.PA453
*Aparatermes cingulatus*	KY683194	UF.SA252
*Aparatermes cingulatus*	KY683192	UF.PA591
*Compositermes bani*	KM538651	
*Compositermes vindai*	KM538649	
*Compositermes vindae*	KM538652	
*Disjunctitermes insularis*	KY683195	UF.PU505
*Disjunctitermes insularis*	KY683188	UF.GU753
*Disjunctitermes insularis*	KY683199	UF.GU788
*Grigiotermes hageni*	KY683196	UF.PA532
*Grigiotermes hageni*	KT215781	
*Grigiotermes hageni*	KY683200	BO241
*Heterotermes crinitus*	KF430191	
*Humutermes krishnai*	KT215787	
*Hydrecotermes kawaii*	KT215788	
*Longustitermes manni*	KF430187	
*Longustitermes manni*	HQ398186	
*Longustitermes manni*	KF430083	
*Macrotermes bellicosus*	AY127702	
*Nasutitermes octopilis*	KF430192	
*Patawatermes turricola*	KY683191	UF.PU597
*Patawatermes turricola*	KY683189	UF.PA1086
*Patawatermes nigripunctatus*	KY683186	UF.EC437
*Patawatermes nigripunctatus*	KT215786	
*Rubeotermes jheringi*	KF430151	
*Rubeotermes jheringi*	KT215778	
*Ruptitermes reconditus*	KM538647	
*Silvestritermes minutus*	KT215789	
*Syntermes grandis*	EU253863	
*Termes hispaniolae*	FJ802753	
Tetimatermes *sp.*	KY683198	UF.SA448

All sequences were aligned using the MUSCLE algorithm in Geneious v6.1.6 (Biomatters Ltd., Auckland, New Zealand). A phylogenetic analysis was conducted under Bayesian inference (BI) with *Heterotermes crinitus* as the outgroup. The substitution model (GTR+I+G) was selected through the Akaike Information Criterion (AIC) with the jModelTest2 (Darriba et al. 2012). The XML input file was generated with BEAUti 1.8.0, and the BI was performed with BEAST 1.8.0 (Drummond et al. 2012). A Yule speciation process with a random starting tree and relaxed molecular clock was used as tree priors. Final Markov chain Monte Carlo (MCMC) searches were conducted for 15,000,000 generations. Convergence and stationarity were assessed with Tracer 1.5 (Rambaut et al. 2014) and the first 150 trees were discarded as burn-in with TreeAnnotator 1.8.0 and visualized using FigTree 1.3.1.

The spatiotemporal spread of *D. insularis* was simulated using methods and biological parameters as described in Tonini et al. 2014. The mean flight distance was defined as 100 meters for this small forest species.

## Taxonomy

### Disjunctitermes

Taxon classificationAnimaliaORDOFAMILIA

Scheffrahn gen. n.

http://zoobank.org/86068307-7A76-4DBF-A369-0B3AC46DD82E

**Figure 1.**
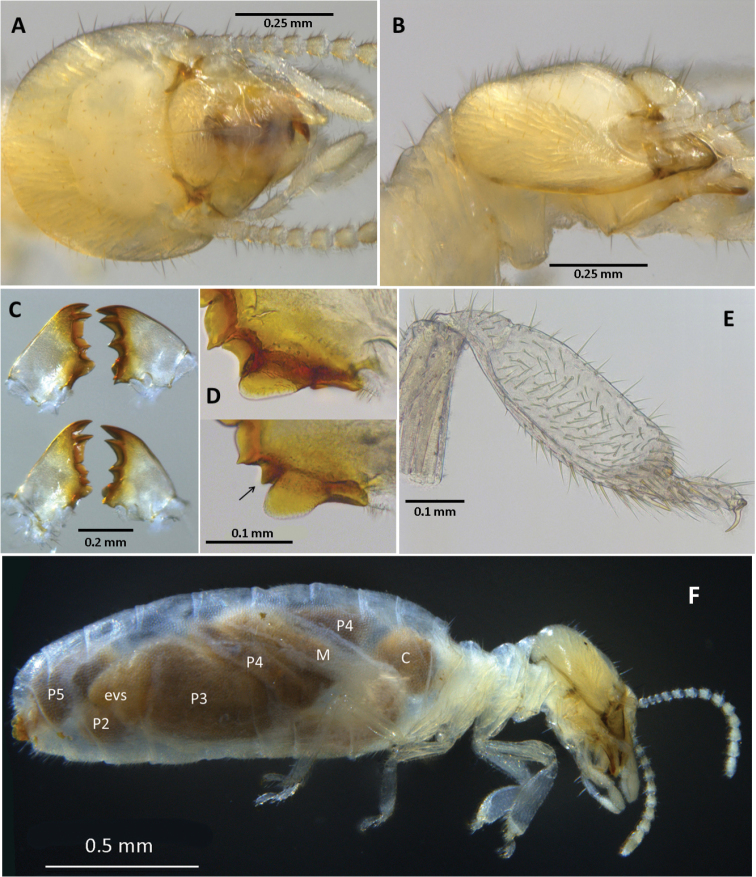
Dorsal (**A**) and lateral (**B**) views of the *Disjunctitermes insularis* worker head capsule **C** Dorsal views of newly molted worker mandibles of *Anoplotermes banksi* Emerson (top) and *D. insularis* (bottom) **D** Ventral views of the molar portion of the left mandibles of newly molted workers of *A. banksi* (top) and *D. insularis* (bottom) **E** Right fore-tibia, and **F** right lateral view of *D. insularis* worker.

**Figure 2.**
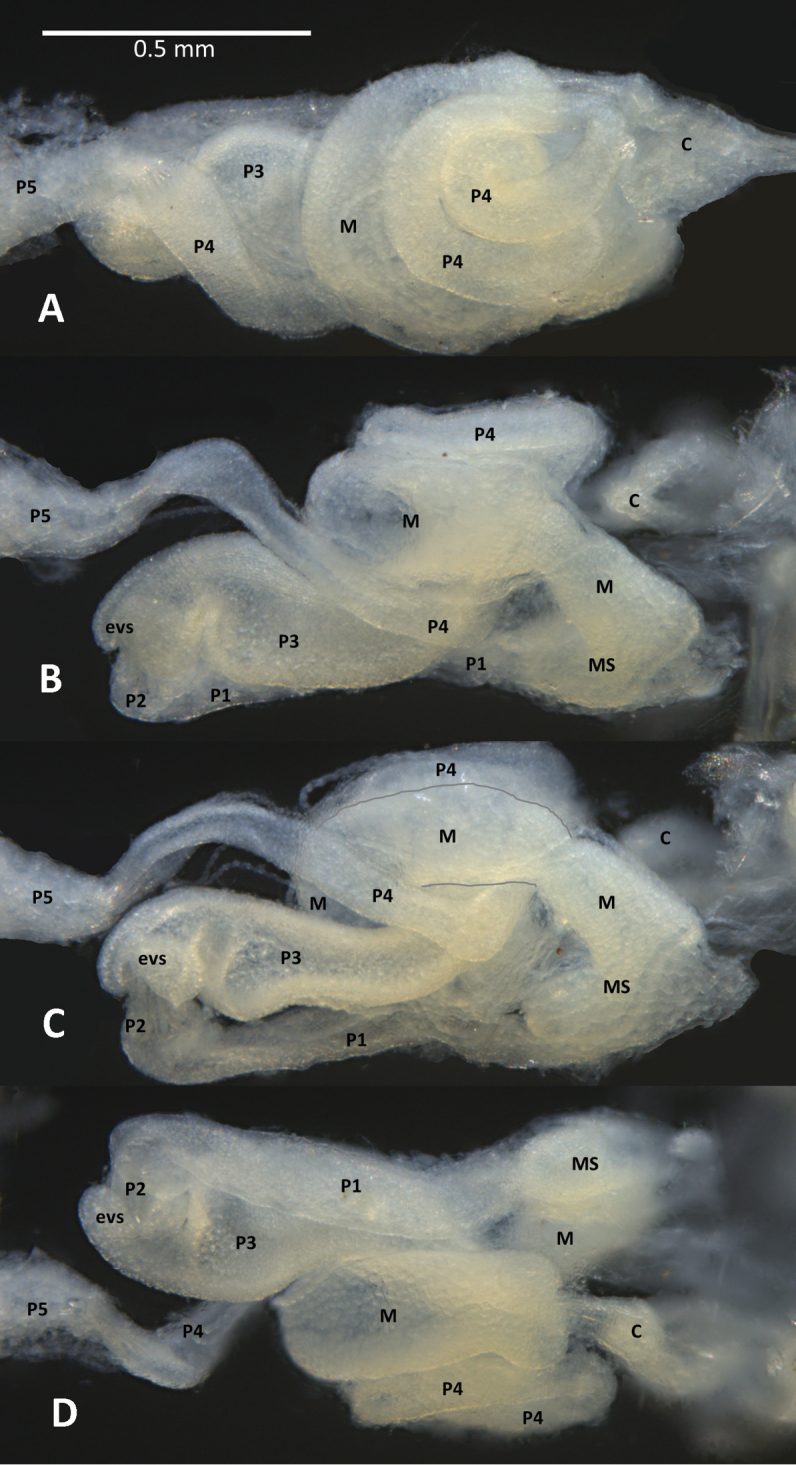
Dorsal (**A**), right (**B**), ventral (**C**), and left (**D**) views of a newly molted, unfed *Disjunctitermes insularis* worker. Abbreviations: C, crop;evs, enteric valve seating; M, mesenteron; MS, mixed segment; P1, P2, P3, P4 and P5 proctodeal segments 1-5, respectively.

**Figure 3.**
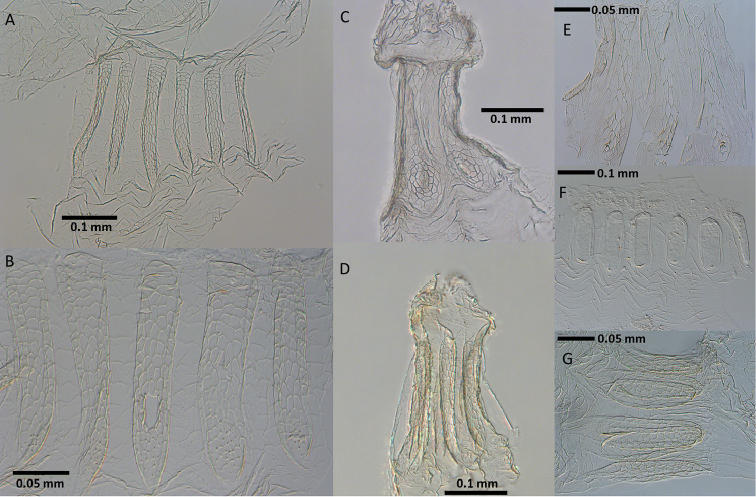
Enteric valve morphology of *Disjunctitermes insularis* worker not fully stretched laterally (**A**) and fully stretched laterally showing five of six pads (**B** center pad with small tear). Whole EV mounts of *A. banksi* (**C**) and *D. insularis* (**D**) with posterior ends at top. Enteric valves of *A. pacificus* (E, 3 pads shown) , *Hydrecotermes arienesho* (F), and *H. kawaii*, whole mount (**G**).

Figs 1 , 2 , 3 , Table 2

#### Type species.

Disjunctitermes insularis sp. n.

#### Diagnosis.

*Disjunctitermes* is one of the described Neotropical apicotermitines that, along with *Anoplotermes banksi*, *A. pacificus*, and *Hydrecotermes* spp., possess strongly inflated fore tibia and lack spiny sclerotized enteric valves. *Disjunctitermes* is closest to *A. banksi*, but can be distinguished from the latter by the subsidiary tooth on the left mandible, the larger EV seating and the more truncate terminus of P2 (Fig. 3C, D). *Hydrecotermes* lacks a spheroidal mesenteric tongue.

#### Imago.

Unknown.

#### Worker

(Figs 1–3, Table 2). Monomorphic, small. Head capsule yellowish, covered with about 100 setae of varying length. Postclypeus moderately inflated, fontanelle barely discernable. Antennae with 14 articles. Left mandible with apical and first marginal teeth well separated, long, and projecting well beyond line formed by third marginal tooth and molar prominence. A subsidiary (fourth) marginal tooth visible above molar prominence in both dorsal (Fig. 1C, bottom) and ventral (Fig. 1D, bottom) views. Right mandible with apical tooth much longer than first marginal; third marginal nearly symmetrical. Fore-tibia strongly inflated; about three times longer than at its widest (median) point. Mesenteric tongue spheroidal (Fig. 2C). P2 entering through large, robustly trilobed EV seating (two lobes prominently visible through integument, Figs 1F, 2C). Enteric valve morphology consists of six elongate, inflated pads (Fig. 3A, B) that face the valve lumen (Fig. 3D). The posterior end of the P2, containing the EV, with truncate terminus projecting about half way into EV seating.

**Table 2.** Measurements (mm) of 12 workers from each of 11 colonies of *D. insularis*.

Colony	Head length to end of postclypeus	Postclypeal length	Max. head width	Pronotal width	Hind tibia length	Fore-tibia width: length ratio
**Holotype**	0.61	0.14	0.64	0.39	0.49	0.29
**GU105**	0.59–0.66	0.13–0.16	0.64–0.69	0.36–0.41	0.48–0.52	0.26–0.31
**GU106**	0.64–0.69	0.14–0.18	0.66–0.69	0.39–0.42	0.48–0.52	0.26–0.33
**GU753**	0.63–0.66	0.14–0.18	0.65–0.69	0.37–0.42	0.48–0.56	0.30–0.36
**GU754**	0.59–0.67	0.13–0.16	0.65–0.69	0.37–0.43	0.46–0.52	0.27–0.35
**GU783**	0.60–0.66	0.14–0.16	0.64–0.66	0.36–0.42	0.48–0.54	0.26–0.35
**GU784**	0.61–0.64	0.13–0.14	0.64–0.67	0.38–0.40	0.49–0.52	0.28–0.33
**GU785**	0.61–0.64	0.13–0.15	0.64–0.67	0.39–0.42	0.46–0.52	0.28–0.34
**GU786**	0.62–0.66	0.14–0.17	0.64–0.70	0.38–0.41	0.49–0.52	.0.28–0.34
**GU787**	0.60–0.64	0.14–0.17	0.65–0.68	0..38–0.40	0.49–0.51	0.28–0.35
**GU788**	0.59–0.63	0.13–0.18	0.63–0.65	0.38–0.42	0.46–0.50	0.28–0.31
**PU505**	0.58–0.64	0.15–0.18	0.64–0.67	0.38–0.42	0.48–0.52	0.28–0.36
**Range (n=132)**	0.58–0.67	0.13–0.18	0.63–0.70	0.36–0.43	0.46–0.56	0.26–0.36

#### Etymology.

**Figure 4.**
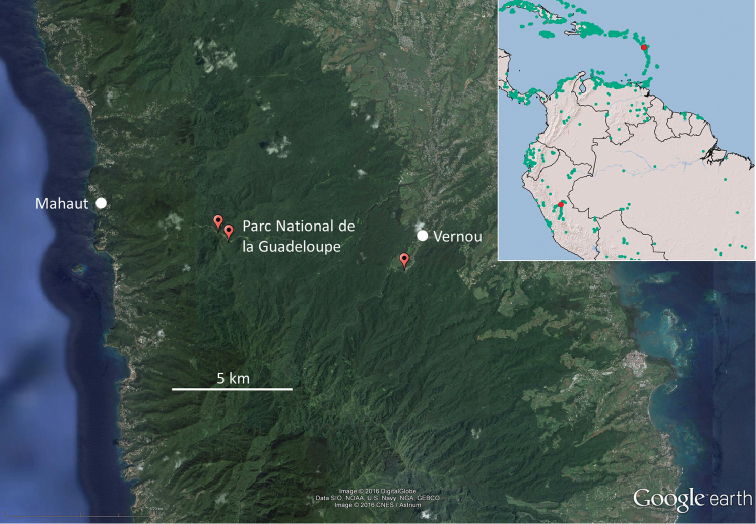
Type localities (red dots) for *D. insularis* on Basse-Terre, Guadeloupe. Inset shows the Guadeloupe and Peru localities (red dots) and all other termite collecting localities in the UF database (green dots) where *D. insularis* was not found.

The genus name is derived from its current, widely disjunct distribution on Guadeloupe and Peru (Fig. 4, inset)

### Disjunctitermes insularis

Taxon classificationAnimaliaORDOFAMILIA

Scheffrahn sp. n.

http://zoobank.org/975729E6-5A94-4DFC-9E00-162E50082D5E

#### Material examined.

Holotype: labelled “(UF code GU 105) GUADELOUPE Basse-Terre, Trail Mamelles de Petite Bourg. Parc Nat., undisturbed forest, 16.1778; -61.7321, 23MAY99, col. Chase, Krececk, Maharajh, Mangold, and Scheffrahn. Paratype colonies (the holotype is kept in the same vial as the paratypes): GUADELOUPE, Basse-Terre 16.1778; -61.7321, 23MAY1999 (GU106), 12 workers; 16.1814; -61.7361, 29MAY1999 (GU753), 12 workers; 16.1814; -73.61, 29MAY1999 (GU754), 11 workers; 16.1674; -61.6644, 29MAY1999 (GU783), 12 workers; 16.1674; -61.6644, 29MAY1999 (GU784), 12 workers; 16.1674; -61.6644, 29MAY1999 (GU785), 12 workers; 16.1674; -61.6644, 29MAY1999, (GU786), 12 workers; 16.1674; -61.6644, 29MAY1999, (GU787), 12 workers; 16.1674; -61.6644, 29MAY1999, (GU788), 12 workers. PERU, 6 km S von Humboldt, disturbed forest, -8.8769; -75.0465, 28MAY2014 (PU505), 12 workers, col. Carrijo, Chase, Constantino, Mangold, Mullins, Křeček, Kuswanto, Nishimura, and Scheffrahn. All material housed at the University of Florida Termite Collection in Davie, Florida. Collection sites are mapped in Fig. 4.

#### Diagnosis.

See also comparison for *Disjunctitermes* above. The EV pads of *D. insularis* differ from those of the four other described species with unarmed EV as follows (Fig. 3): each pad of *A. banksi* is vase-shaped, with a narrow posterior end that widens into an oval base reminiscent of an orb-weaving spider web (Fig. 3C); the *A. pacificus* pads are shaped similarly to those of *A. banksi* but are less concentric and are adorned with a few unsclerotized spines (Fig. 3E); while the pads of *H. arienesho* and *H. kawaii* are ovoid in shape (Figs 3F and 3G, respectively).

#### Imago.

Unknown.

#### Worker

(Figs 1–3, Table 2). See *Disjunctitermes* gen. n. description above. EV devoid of sclerotized spiny armature. Pads about six times longer than wide; slight difference in length when stretched horizontally. Anterior fourth of each pad composed of about 10-20 ovoid scales each with one point facing posteriorly. Posterior portion of pads truncate with about 30-50 polygonal scales adorned with fringes on their posterior margins. Cuticle between pads with about 15-20 faint arching ridges; ridges fringed posteriorly.

#### Etymology.

The species name is derived from its unexpected island locality.

#### Habitat and biology.

Workers were collected in foraging groups under rocks and stones in rainforest habitats. Like many New World Apicotermitinae, *D. insularis* does not build any above-ground structures. Mature worker gut contents confirm that they feed on the organic fraction of soil.

#### Molecular phylogeny.

**Figure 5.**
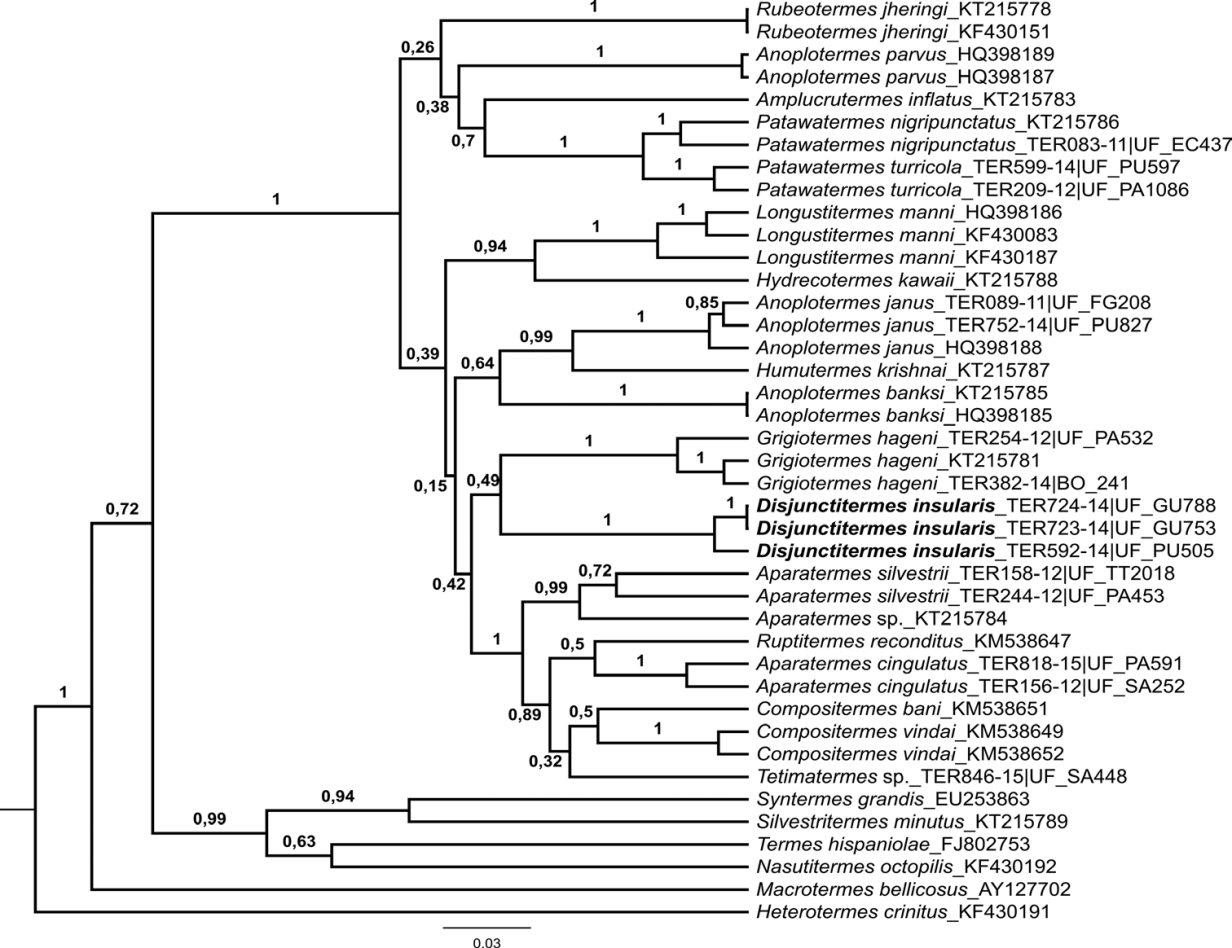
Bayesian phylogeny of all described soldierless New World genera using the mitochondrial CO1 barcode gene showing posterior probabilities. Tree rooted on terminal *Heterotermes crinitus*.

The molecular phylogeny performed with the mitochondrial gene COI clearly clustered *D. insularis* specimens from Guadeloupe and Peru, as well as specimens belonging to the same species of other genera (Fig. 5). However, the phylogeny showed low resolution in the relationships between the Apicotermitinae genera.

#### Dispersal rate on land.

**Figure 6.**
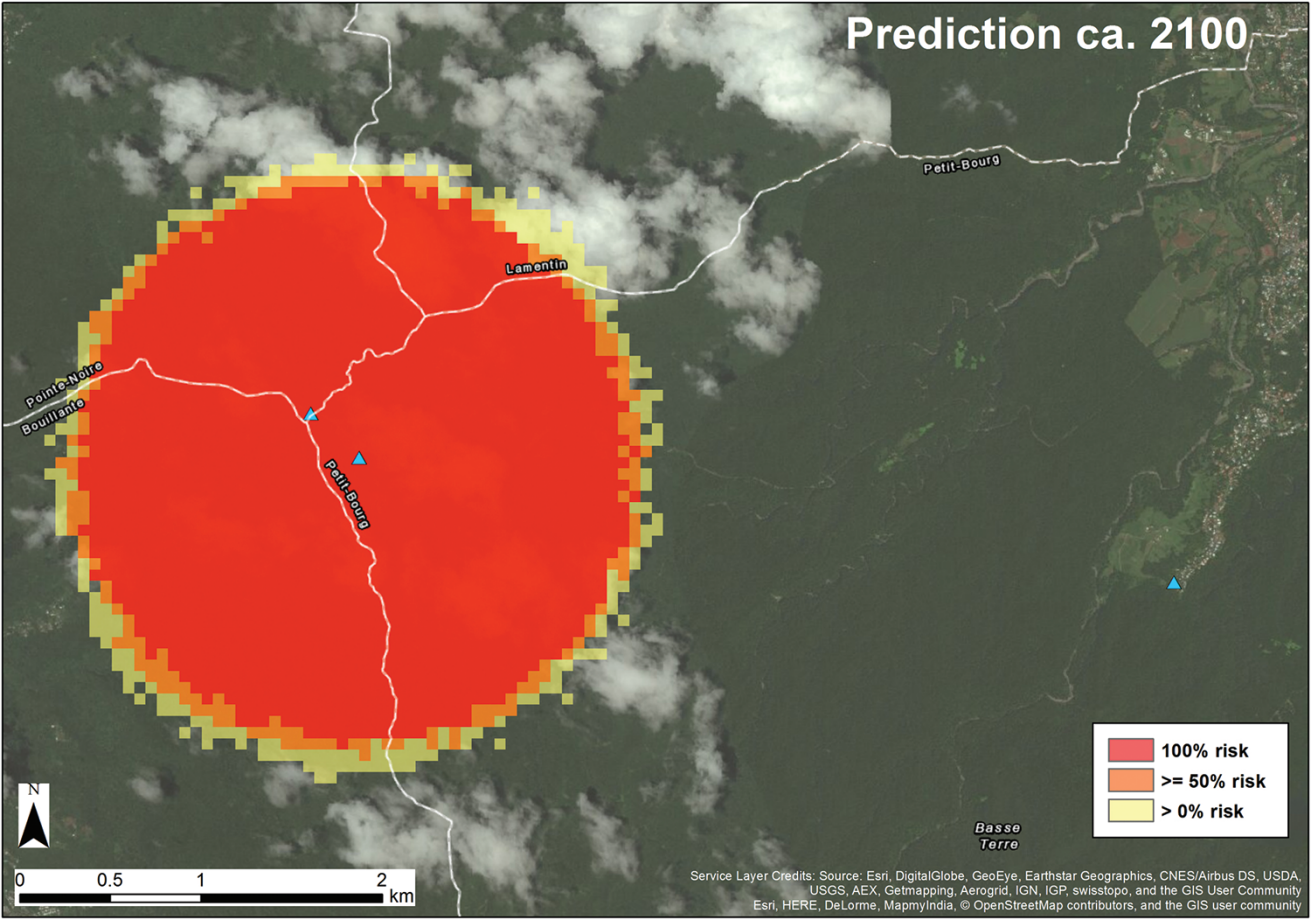
An 85-year stochastic lattice-based model simulation of *Disjunctitermes insularis* spread from a single founder point locality on Basse-Terre, Guadeloupe.

Starting from a single founder location, the stochastic spread models predicts a 2,778-meter spread over 85 years (Fig. 6) or about 265 years to reach the ca. 8 km expanse between the easternmost and westernmost collection localities (Fig. 4). This suggests a very remote possibility that a single human transport event delivered *D. insularis* to Guadeloupe which would have taken place at a time when French colonization of Basse-Terre was limited to the coast (Hoy 1961). It is far more likely, however, that *D. insularis* reached Guadeloupe via a natural overwater dispersal event (De Queiroz 2005) during pre-Colombian times.

#### Taxonomic correction.

Darlington (1992) reported a *Neotermes* sp. between 600-1000 m on Basse-Terre which we found to be *Comatermes perfectus* (Hagen).

## Discussion

Before 1960, all New World soldierless termites were described from the imago caste and placed in the genus *Anoplotermes* (Krishna 2013). Using Sands (1972) descriptive methods for Old World taxa, Mathews (1977) was the first to adopt internal worker characters, including the EV (Grassé and Noirot 1954), for New World soldierless termites (*Anoplotermes, Grigiotermes, and Ruptitermes*). As imagos are sometimes difficult to collect, Fontes (1986) was the first to describe a neotropical soldierless taxon based only on diagnostic characters of the worker caste (*Tetimatermes*, fore tibia), followed by Scheffrahn 2013 (*Compositermes*, EV), and Bourguignon et al. 2016a (*Amplucrutermes*; fore tibia, EV, and gene sequence). Given the robust morphology of the EV of soldierless and other soil-feeding termites, and guidance from sequence reconstructions (Bourguignon et al. 2013, Bourguignon et al. 2016a, Carrijo et al. 2015), *Disjunctitermes insularis* is the newest worker-based soldierless taxon. The senior author has participated in over 75 termite diversity expeditions from 1990-2014 and recognizes about 40 undescribed soldierless genera from the Neotropics based, in large part, on EV morphology and CO1 sequences. All specimens are housed in the UF collection.

Short overwater or vicariant dispersal transported the Apicotermitinae to continental shelf islands such as Cuba and the Bahamas (Scheffrahn et al. 2006) or Trinidad and Tobago (Scheffrahn, unpublished) during low sea level stands of the Late Pleistocene. As with all the Termitidae, the Apicotermitinae diversified some 40 mya (Engel et al. 2009) after the continents were separated by vast oceans (Scotese 2004). Therefore, the more basal Old World Apicotermitinae probably arrived in the New World via a single transoceanic dispersal event (Bourguignon et al. 2017).

To our knowledge, *D. insularis* is the only soldierless or soil-feeding termite inhabiting a deep-water (>950 m for Guadeloupe) island. Snyder (1949) listed *Termes morio* Latreille from Martinique as a synonym of *Anoplotermes meridianus* Emerson, however, Emerson (1925) found that *T. morio*, as described by Latreille, is actually *Nasutitermes costalis* (=*corniger*). Basse-Terre Guadeloupe, part of the Antillean volcanic arc, was formed 2.8 mya (Samper et al. 2007). Our discovery of *D. insularis* on Basse-Terre is also the first record of a non-wood-feeding termite on a deep-water island (cf. Krishna et al. 2013 volumes 4–6). There is no record of anthropogenic transport of any non-wood-feeding termite (Evans 2011) and the localities of *D. insularis* (Fig. 4) are mountainous with rocky soil that is ill-suited for agriculture and development (Hoy 1961). Even today there are no villages or towns in the climax forests between Mahaut and Vernou (Fig. 4).

We surmise that the establishment of *D. insularis* was the result of a natural overwater dispersal event from the mainland Neotropics followed by natural spread presumably across the entire forested interior of Guadeloupe which we incompletely surveyed (Fig. 4). Although known only from a single Amazonian locality in Peru, *D. insularis* is probably widespread in the Neotropics as has been the case for many other soldierless species (Bourguignon et al. 2010, 2016a; Scheffrahn 2013).

## Supplementary Material

XML Treatment for Disjunctitermes

XML Treatment for Disjunctitermes insularis
